# Spatiotemporal Variation and Risk Assessment of Pesticides in Water of the Lower Catchment Basin of Acheloos River, Western Greece

**DOI:** 10.1155/2013/231610

**Published:** 2013-12-28

**Authors:** Nikolaos Stamatis, Dimitra Hela, Vassilios Triantafyllidis, Ioannis Konstantinou

**Affiliations:** ^1^Department of Environmental and Natural Resources Management, University of Patras, Seferi 2, 30100 Agrinio, Greece; ^2^Department of Aquaculture and Fisheries, Faculty of Agricultural Technology, Technological Education Institute of Messolonghi, 30200 Messolonghi, Greece; ^3^Department of Agricultural Enterprise and Food Management, University of Patras, Seferi 2, 30100 Agrinio, Greece

## Abstract

A three-year monitoring survey (March 2005–February 2008) was conducted to investigate, on monthly basis, the presence of thirty pesticides belonging to various categories and metabolites, in Acheloos River (Western Greece), one of the most important water resources in Greece. Six sampling stations along the river were established. Water analyses were performed using solid-phase extraction combined with gas chromatography with flame thermionic detector and mass spectrometry. Statistical analysis using one-way ANOVA and Duncan's multiple range test (*P* < 0.05) was used to compare annual mean concentrations of pesticides, seasonal and spatial distribution. In general, the highest mean concentrations of the pesticides were recorded at the three stations downstream. The greatest average concentrations were determined during spring and summer in agreement with the pesticide application period. The observed lower concentrations after 2006 reflect the land-use change because of the elimination of tobacco, the main cultivation of the area for many decades. The compounds most frequently detected were diazinon (78.6%), DEA (69.3%), and fenthion (52.6%). Environmental risk assessment using risk quotient (RQ) approach showed high risk for six insecticides in 2005 and one in 2007. A compliance with the European Environmental Quality Standards (EQS) was observed for the priority pesticides.

## 1. Introduction 

The occurrence of pesticides and their conversion products in aquatic systems is one of the major environmental problems worldwide. Their widespread use combined with overapplication, accidental spills, runoff from cultivated areas, and faulty waste disposal creates environmental pollution concerns [1]. During the last three decades, pesticide usage for agricultural and nonagricultural purposes has increased dramatically and has resulted in the presence of their residues in various environmental matrices. Although there are many significant practices to manage point-source pollution, very little progress has been achieved in facing nonpoint source pollution, especially of surface waters, due to seasonal variations, inherent problems, and multiplicity of the processes [2]. Surface runoff is the main way for pesticides moving from agricultural fields to surface waters [3]. Several factors, such as the topography and the weather of the studying area, soil characteristics, agricultural practices, and chemical and environmental properties of individual pesticides, play the major roles for the amount of pesticides lost from fields and transported to aquatic environment [4]. The temporal and spatial distribution of pesticide concentrations depends on all these factors, but it is difficult to be predicted [5].

In recent years, analytical methods based on solid-phase extraction (SPE), solid-phase microextraction (SPME), and liquid-phase microextraction (LPME) have been used. These techniques are inexpensive, as they require common laboratory equipment and very small amounts of organic solvents. Besides, they offer the advantages of simplicity, shorter analysis time, and less interferences. Solid-phase extraction has been widely used as an alternative method for the isolation and the preconcentration of many organic compounds, including pesticides, and has been extensively applied to the extraction of such pollutants from water samples [2, 6, 7].

The great majority of extraction techniques for pesticides are followed by gas chromatography (GC) or high performance liquid chromatography (HPLC) because of the complexity of the matrices and the low concentrations of pesticides in natural water samples (usually ng L^−1^). HPLC methods are more suitable for determining thermally labile and polar pesticides. Both chromatographic techniques methods are very efficient for determining environmental pesticide residues with high resolution and sensitivity using specific detectors or coupled with mass spectrometry.

The determination of pesticides in water samples, such as river, lake, and sea water, is necessary in order to verify whether inadmissible levels are present. In the last decade, emphasis has been given to pesticides with higher polarity and lower persistence. Due to their enhanced solubility, modern pesticides are able to reach the surface water through agricultural runoff and leaching to ground water [8]. Numerous studies have revealed the widespread occurrence of pesticides in European and American fresh surface waters and ground waters [9].

Pesticide residues have been detected also in surface water, ground water, and drinking water samples across Greece indicating that some major water resources are contaminated [9]. These detections are the result of extensive regional and nationwide studies often using analysis at very high sensitivity. According to the occurrence and concentration range of pesticides detected in rivers in Greece, two main groups can be divided. The first one includes pesticides that are occasionally detected sharing one or more of the following characteristics: low application rates, usage in limited geographical areas, short soil lifetimes, short aquatic lifetimes, and lower run-off hazard. The other group consists of the compounds that are found frequently in Greek surface waters and represent seasonally increased concentrations (i.e., atrazine, alachlor, and diazinon). Characteristic properties of the above compounds are: higher application rates, widespread usage, higher hazard due to runoff, and longer aquatic half-lives [9].

The data available in running literature concerning river water pollution by pesticides in Greece regard a number of principal rivers that are draining mainly agricultural areas. Most studies on pesticide monitoring of Greek freshwater resources have been summarized in a previously published review by the authors [9]. Most of the major Greek rivers like Aliakmon [10], Loudias [11], Axios [12], Pinios [13], Kalamas [14], Mornos [15], and Evrotas [16] and major lakes like Trichonida [13], Marathonas [16], Pamvotida [14], Volvi, Vistonida, and Prespa [17] have been monitored for pesticide residues. In addition, the existing information covers the last two decades but only few systematic monitoring studies that include several pesticide categories have been published for rivers such as Aliakmon, Axios, Loudias, Louros, Arachthos, and Kalamas. However, no information was available for the pesticide contamination of the River Acheloos, one of the most important Greek rivers; although that studies on physicochemical parameters, nutrients, organic carbon and other hazardous compounds like metals and hydrocarbons have been reported [18–21].

This work presents for the first time a seasonal and spatial study on the variation and distribution of pesticides in the water of Acheloos River for a three-year monitoring period (2005–2008). Solid-phase extraction followed by gas chromatography with flame thermionic detector (FTD) and mass spectrometry (MS) were applied for the screening of thirty pesticides and metabolites. Statistical analysis of the results was performed in order to investigate significant differences in pesticide concentration among years, seasons, and sampling sites. An important objective of the study was also to evaluate the effect of land-use changes after the considerable reduction of tobacco, which was the main cultivation of the region for a long period in the past. In addition, the point source of pesticide pollution resulting from the outflow of wastewater treatment plant (WWTP) of Agrinio was investigated. Finally, the risk assessment and compliance with the established environmental quality standards according to the Water Framework Directive (2000/60/EC) are discussed.

## 2. Experimental 

### 2.1. Area Description and Sampling Site

Acheloos River, located in the southwestern part of the country, is one of the most important rivers in Greece, the first in water contribution, and the second in length, found in the Greek territory. The river springs from Pindos Mountains at an altitude of 1,700 m and crosses a distance of 235 km before it meets the Ionian Sea. Its yearly outflow is estimated to be 7.8·10^9^ m^3^. The average precipitation in the basin is approximately 1,380 mm/year. The rainy season lasts from November till February and driest months are July and August. The drainage basin of Acheloos covers a total area of 6329 km^2^ and includes three major subcatchments, the upper part (1100 km^2^), the middle part (3250 km^2^), and the lower part (1979 km^2^) of the basin. The shape of its basin is oblong with a maximum axis of 147 km length and 63 km width. The upper part of the basin has major tributaries such as Karpenisiotis, Trikeriotis, and Agrafiotis rivers and includes mountainous terrain (mean catchment altitude 840 m). In the middle subcatchment four large hydroelectric dams and an irrigation dam (Tavropos, Kremasta, Kastraki, and Stratos) that form artificial lakes are situated. Detailed morphometric characteristics of the Acheloos reservoirs are published elsewhere [18]. The lower part includes the Trichonida, Lysimachia, Amvrakia, and Ozeros natural lakes, the allouvial plain and the Messolonghi, Etoliko, and Klisova lagoons [18]. The delta plain, which covers an area of 300 km^2^ [22] consists of a large network of irrigation channels.

The Acheloos estuary is of high environmental importance as it affects the distributions of nutrients in the entire Northwestern section of Patraikos Gulf and in the nearshore part of the Ionian Sea [20]. In addition, the ecological importance of the estuary is high as it is connected to coastal lagoons which are under the protection of the Ramsar convention. Finally, the delta plain belongs to the Natura 2000 sites.

The Acheloos River water is used in the agriculture and the generation of electricity. The watershed is not industrialized and agriculture contributes about 45% of the average income for the region. Acheloos River receives the land washout and runoff of a relatively large cultivated area situated mostly in the middle and lower part of its basin causing serious nonpoint source pollution. In particular in the lower part of the river delta agricultural land covers 41% of the area [18].

**Figure 1:**
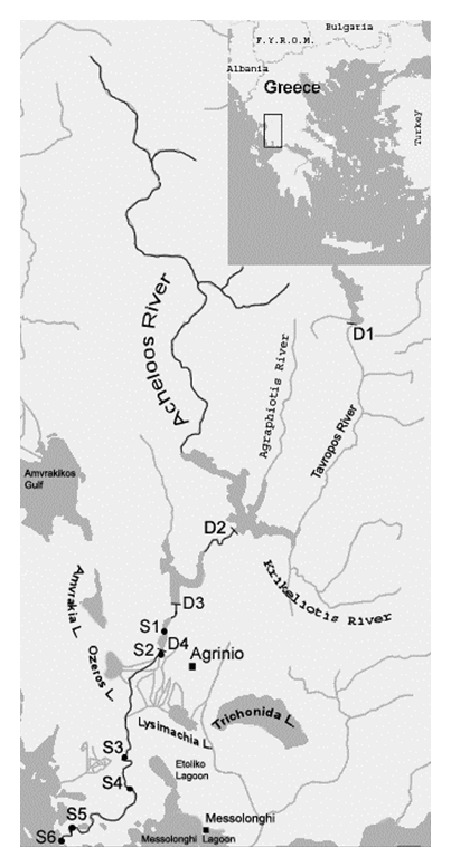
Map of the six sampling sites: S_1_: bridge at village Matsouki, S_2_: after the dam of Stratos, S_3_: after the outflow of WWTP of Agrinio, S_4_: bridge between villages Neochori and Katochi, S_5_: 5 km before the river mouth, S_6_: the river mouth, and four hydroelectric dams (D_1_: Tavropos, D_2_: Kremasta, D_3_: Kastraki, and D_4_: Stratos).

The monitoring of the present study is focused on the middle and lower part of the river basin while the upper part is excluded due to the mountainous relief and the absence of significant agricultural activity. Six (S_1_–S_6_) stations were sampled along the main flow and estuary of the river (Figure 1). The selected sampling stations are representative of the major freshwater inputs into the river and, as such, represent possible sources of pesticide discharge into the river: the bridge at village Matsouki (S_1_), the site after the dam of Stratos (S_2_), after the discharge of WWTP of Agrinio city (S_3_), the bridge between villages Neochori and Katochi (S_4_), 5 km before the river mouth (S_5_), and at the river mouth (S_6_). Dams affect the flow of Acheloos River more than the seasonal rainfall (Figure 1). Due to loss of water that occurs along river's flow in the wide irrigation network at the lower part of the basin, the basic maintenance flow in the estuaries was reported to vary from a mean monthly flow of 17.8 (July) to 34.7 m^3^s^−l^ (January) [23]. Composite hydrographs for the mean monthly discharge of Acheloos, upstream and downstream of the reservoirs, for 22-year time series (1980–1999) are reported elsewhere [18]. Finally, Acheloos River receives the treated effluents of the wastewater treatment plant (WWTP) of Agrinio city, the larger urban center in the area. Details on the description of WWTP can be found elsewhere [24, 25].

### 2.2. Chemicals

All pesticide standards (purity >98%) were purchased from Riedel-de-Haën (Seelze, Germany). All solvents used (acetone, LC-grade water, ethyl acetate, and methanol) were pesticide residue analysis grade from Merck (Darmstadt, Germany). Primary stock standard solutions of the target pesticides were prepared individually in methanol at a concentration of 200 ng/mL and stored at −18°C. The working solutions of the mixtures at various concentrations were prepared by appropriate dilution of the stock solutions and were stored at 4°C in the dark. Calibration standards were renewed every week. Oasis HLB (divinylbenzene/N-vinylpyrrolidone copolymer) cartridges (200 mg, 6 mL) from Waters (Mildford, MA, USA) were used for water samples extraction.

### 2.3. Water Sampling

Water samples (2.5 L) were taken using polypropylene water samplers (Windaus-Labortechnik) at a depth of 1 m below the water surface. Water samples were collected monthly between March 2005 and February 2008. They were collected in precleaned amber glass bottles and transported to the laboratory under cool conditions. Upon arrival to the laboratory within 6 h of collection, the samples were filtered through filter paper (Whatman, USA) to eliminate particulate matter and other suspended solid matter and then stored in the dark at 4°C in a cold room. Further extraction of the samples was carried out within 24 h of collection to keep microbial degradation to a minimum. The target analytes included thirty pesticides and metabolites selected on the basis that they have been previously reported in environmental surface waters of Greece and other European countries and on data for the agricultural application in the basin. Seven of them belong to the priority pollutants of Annex I, Directive 2008/105/EC.

### 2.4. Sample Extraction and Chromatographic Analyses

The extraction and sample preparation of the water samples are based on offline solid-phase extraction (SPE). SPE was performed using a 12-fold vacuum extraction box (Visiprep, Supelco, Bellefonte, PA, USA) fitted on a pump to achieve the appropriate vacuum for the solid phase extraction. Prior to the extraction water samples were allowed to reach room temperature. The SPE cartridges (Oasis HLB) were conditioned with 5 mL of ethyl acetate, 5 mL methanol, and 5 mL LC-grade water at a flow rate of 1 mL min^−1^. Then, water samples were added at a flow rate of 10 mL min^−1^ and finally the cartridges were washed with 6 mL grade water. The cartridges were dried by nitrogen stream for 20 minutes. After sample extraction, the pesticides trapped in the cartridge were collected by using 2 × 5 mL of ethyl acetate as eluting solvent at 1 mL min^−1^. Small quantities of anhydrous Na_2_SO_4_ were added to remove any water content in the sample. The eluate was concentrated to a final volume of 0.2 mL in a gentle stream of nitrogen.

A Shimadzu 17A capillary gas chromatograph equipped with flame thermionic detector (FTD) and Equity-1 column (30 m, 0.25 mm I.D., 0.25 *μ*m) containing dimethylpolysiloxane was used. The column was programmed from 55°C (2 min) to 160°C (10 min) at 5°C min^−1^, to 210°C (20 min) at 5°C min^−1^, and to 270°C (2 min) at 20°C min^−1^. Helium was used as carrier (1.5 mL min^−1^) and make-up gases (40 mL/min), respectively. The detector gases were hydrogen and air at flows of 4 and 120 mL min^−1^, respectively. The detector temperature was set to 290°C and the injector temperature to 220°C. An alkali metallic salt (Rb_2_SO_4_) bonded to a 0.2 mm spiral of platinum wire generated the ions. The splitless mode was used with the valve opened after 60 s. The injection volume was 2 *μ*L. Quantification of pesticides was performed using the internal standard (fenitrothion) method based on peak areas.

Secondary confirmation was performed using a GC-MS, QP-2010 Shimadzu equipped with a soft polar capillary column SPB 5 ms (30 m, 0.25 mm, 0.25 *μ*m), containing 5% phenylpolysiloxane and 95% dimethyl-polysiloxane, used at the following chromatographic conditions: injector temperature 220°C, column program of temperatures 55°C (2 min) to 154°C (3 min) at 3°C min^−1^, to 160°C (7 min) at 1°C min^−1^, to 210°C (4 min) at 5°C min^−1^, and to 270°C (2 min) at 20°C min^−1^. Helium was used as the carrier gas at 67.3 KPa. The ion source and transfer line were kept at 200°C and 310°C, respectively. The quadrupole mass spectrometer was operated in electron impact (EI) ionization mode at 70 eV and monitored ions from *m*/*z* 50 to 450. The splitless mode was used for injection of 2 *μ*L volume, with the valve opened for 30 s. Characteristic ions of the selected pesticides [25, 26] were chosen for screening analysis in selected ion monitoring (SIM) mode.

### 2.5. Quality Control and Treatment of Data

Validation studies of the method were performed using river water. The recovery studies were carried out by spiking three replicates of river samples at the concentration level of 0.1 *μ*g L^−1^. For each pesticide studied the mean recovery value ranged between 70 and 120% while the relative standard deviation was less than 15% [25, 26]. The precision of the method, determined as relative standard deviation (RSD), was obtained from the repeated analysis (*n* = 5) of spiked extracts during the same day (repeatability) and in different days (reproducibility). Repeatability of the method was considered satisfactory with standard deviations from 5 to 16%. The limits of detection (LODs) were determined experimentally from the injection of spiked river samples and calculated using a signal-to-noise ratio (*S*/*N*) = 3. Low LODs were achieved ranging from 2 ng L^−1^ to 15 ng L^−1^ in water samples [25, 26]. The confirmation criteria applied to the target pesticides in the wastewater samples were presence of the three characteristic fragment ions (Table 1) at the correct retention time (±0.05) and with the correct relative ion intensity (±30%).

The results obtained by the three-year monitoring of Acheloos River were statistically analyzed. Values were compared by one-way ANOVA test and mean differences were determined using Duncan's test (*P* < 0.05). In cases that the sample concentrations were below the LOD, a concentration equal to half of the detection limit was used for the calculations. The data were analyzed using the SPSS 15.0 program for Windows.

### 2.6. Calculation of PNECs and Risk Assessment

The environmental risk posed by the studied pesticides on Acheloos River ecosystem was assessed through the calculation of risk quotients (RQ) as described previously [27]. RQ values for aquatic organisms were calculated from the measured environmental concentration (MEC) and the predicted no effect concentration (PNEC) of the pesticides (RQ = MEC/PNEC). In order to overcome the uncertainty of this conservative assessment associated with the accuracy, inherent variability, model errors, and lack of data in the determination of toxicity values, PNEC values were calculated by dividing the lowest long-term NOEC or short-term L(E)C50 (lethal/effect) when NOEC values are lacking, for the most sensitive species by the appropriate assessment factors (AFs) for three trophic levels (fish, zooplankton, and phytoplankton) according to the European Technical Guidance Document [27–29]. The assessment factor can vary depending on the organisms being assessed and whether the toxicity endpoint is acute, based on short-term, lethal, or immobility effects (LC/EC50) or chronic, based on no observed effect (NOEC). According to TGD guidelines [27], an assessment factor of 1000 was used in the cases that at least one short-term assay at one trophic level was available, an assessment factor of 100 was used when data from one long-term assay with either fish or zooplankton were available, and finally assessment factors of 50 and 10 were used in the cases of two and three existing long-term assays, respectively. Ecotoxicological data (Table 6) were obtained from FOOTPRINT pesticide properties database [30], PAN Pesticides Database [31] and other studies containing toxicological data [32]. RQ for each pesticide was calculated using the worst-case scenario; that is, the maximum MEC was used. A commonly used risk ranking criteria were applied: RQ < 0.1 means minimal risk, 0.1 ≤ RQ < 1 means median risk, and RQ ≥ 1 means high risk [33].

## 3. Results and Discussion

### 3.1. Occurrence and Spatiotemporal Variation of Pesticides

Thirty pesticides and metabolites were analyzed in the water samples of Acheloos River. Ten of them were sporadically detected (carbofuran, simazine, pyrimethanil, quinalphos, fenthion sulfoxide, triazophos, azinphos methyl, phosalone, pirimiphos methyl, and tebuconazole); therefore they were not statistically studied. Four herbicides (alachlor, atrazine, S-metolachlor, trifluralin), one metabolite (desethyl atrazine, DEA), nine insecticides (chlorpyrifos, chlorpyrifos methyl, diazinon, dichlorvos, dimethoate, fenthion, methidathion, parathion methyl and pirimiphos methyl), one metabolite (malaoxon), and four fungicides (cyproconazole, penconazole, pyrazophos, and triadimefon), were detected in the water samples during the monitoring period of three years: 2005, 2006, and 2007.

**Figure 2:**
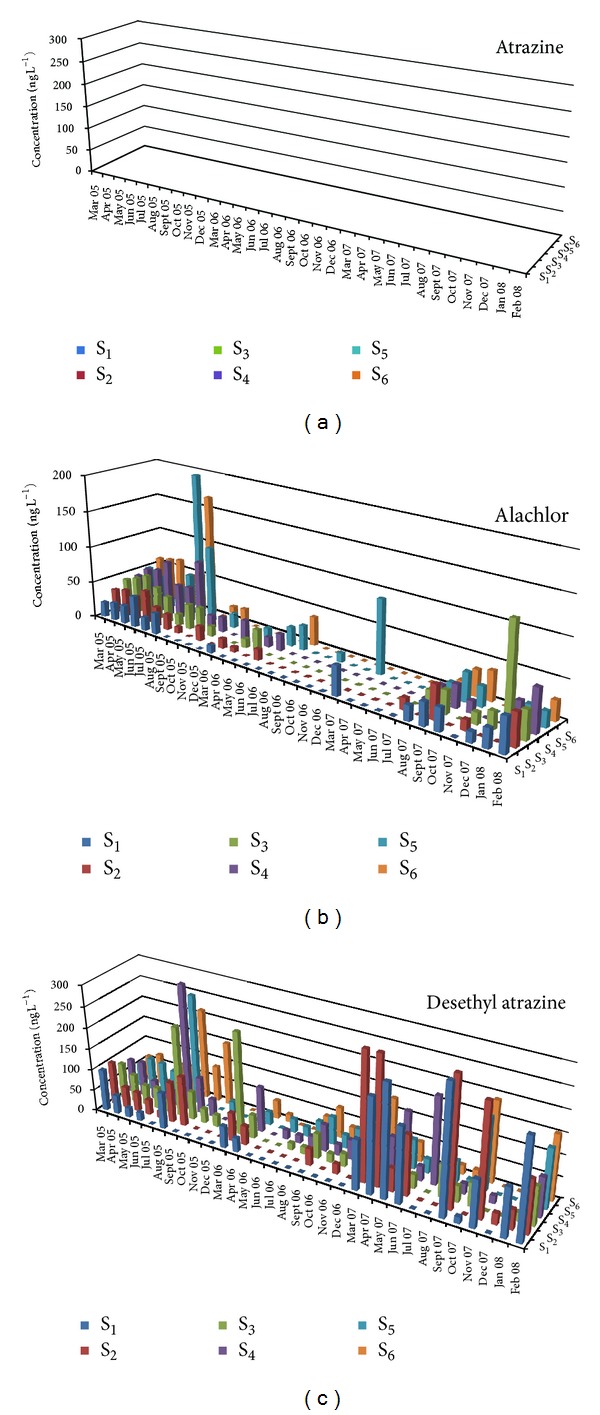
Temporal and spatial distribution of selected herbicide (atrazine (a), alachlor (b)) and metabolites (DEA (c)) concentrations during the sampling period (March 2005–February 2008) for the six sampling sites.

**Figure 3:**
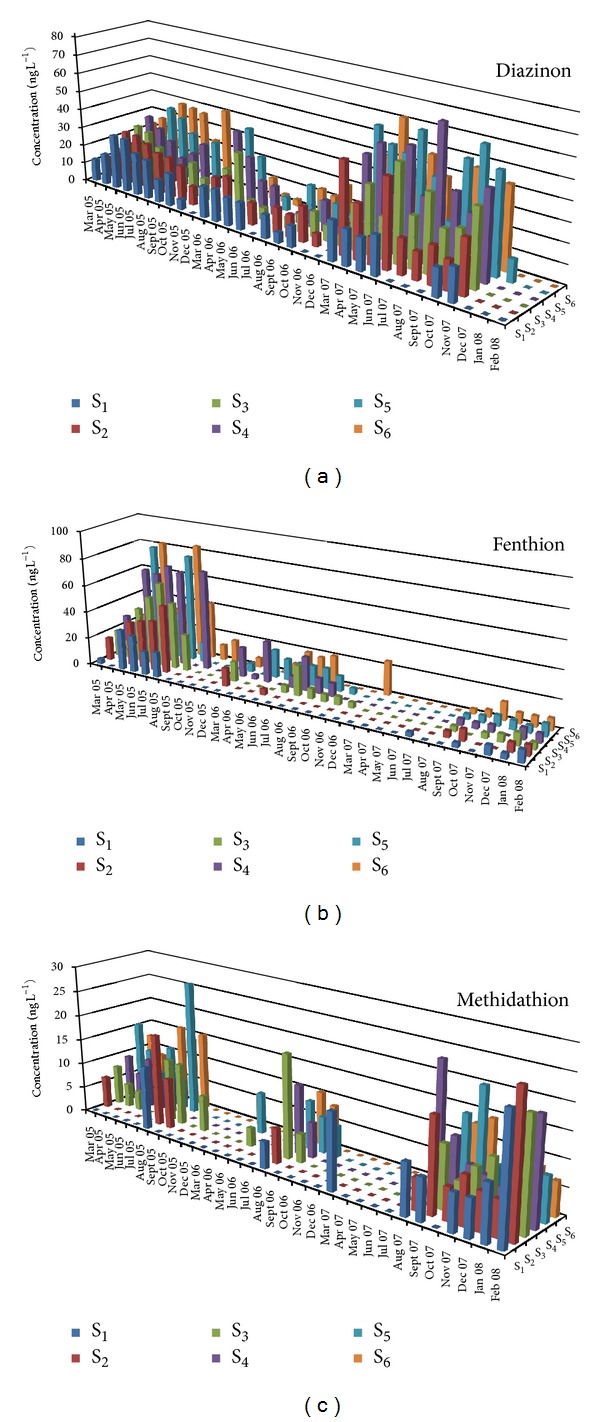
Temporal and spatial distribution of selected insecticide (diazinon (a), fenthion (b), and methidathion (c)) concentrations during the sampling period (March 2005–February 2008) for the six sampling sites.

**Figure 4:**
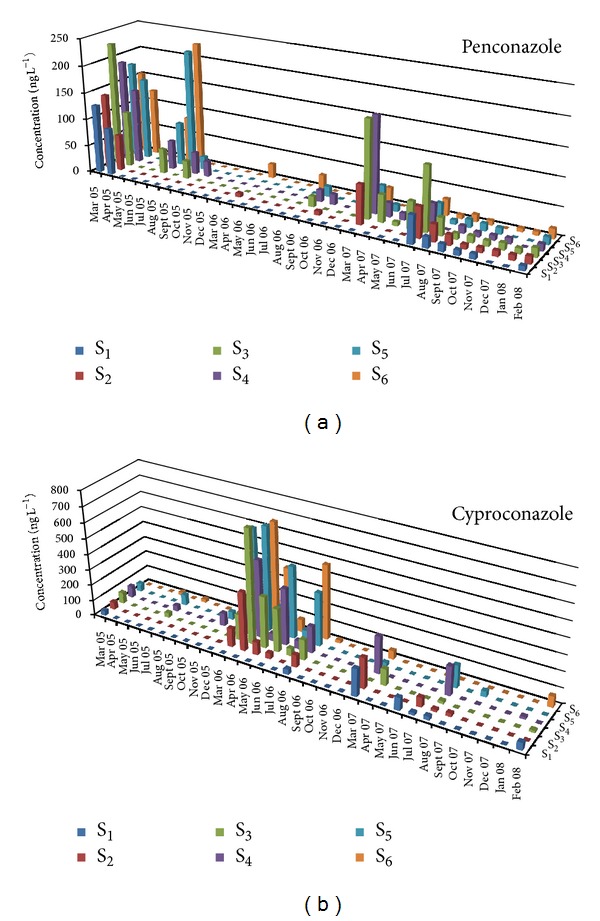
Temporal and spatial distribution of selected fungicide (penconazole (a) and cyproconazole (b)) concentrations during the sampling period (March 2005–February 2008) for the six sampling sites.

Table 1 presents mean, minimum, and maximum concentrations and the percentage frequency of detections of the nineteen selected pesticides for the period 2005–2007 and all stations. The highest frequency of detection was observed for diazinon (78.6%), alachlor (50%), penconazole (43.2%), and DEA (69.3%) for insecticides, herbicides, fungicides, and metabolites, respectively. The above pesticides combine two or more of the following properties: widespread use, high soil and aquatic half-lives, and run-off hazard. They were used in past and recent years in various cultivations in the lower part of Acheloos basin such as corn, olive trees, tobacco, cereals and vegetables. Low detection frequencies were observed for eight pesticides (chlorpyrifos 31.8%, cyproconazole 31.8%, chlorpyrifos methyl 31.3%, parathion methyl 29.7%, trifluralin 28.1%, S-metolachlor 24.5%, dimethoate 21.4%, and pyrazophos 17.2%). These pesticides have one or more of the following properties: small scale use, short soil and aquatic lifetimes, and low run-off hazard. The spatiotemporal variation of the most frequently detected compounds for each pesticide category is presented in Figures 2, 3, and 4.

#### 3.1.1. Spatial Distribution

A general trend for the three categories (herbicides, insecticides, and fungicides) of target compounds was observed in annual average concentrations for the selected sampling stations. Greater values were recorded for the last three stations (S_4_, S_5_, and S_6_) than those for the stations upstream (S_1_, S_2_, and S_3_). However, nonsignificant differences were observed for thirteen of the compounds (Table 2). This reflects the present and past widespread uses of the compounds in most of the cultivations in the lowest Acheloos basin agricultural area (i.e., olives, corn, cotton, alfalfa, citrus, asparagus, vegetable, rice, and grapes). In addition, significantly different mean annual concentrations along stations were observed for pesticides such as chlorpyrifos, fenthion, diazinon, and dichlorvos.

The spatial distribution of annual means for herbicides showed maximum concentrations for atrazine (S_4_: 70.9 ± 29.8 ng L^−1^) and its metabolite DEA (S_4_: 91.1 ± 48.2 ng L^−1^) in 2005, trifluralin (S_6_: 34.8 ± 24.8 ng L^−1^) and DEA (S_3_: 44.8 ± 23.0 ng L^−1^) in 2006, and alachlor (S_5_: 20.9 ± 9.51 ng L^−1^) and DEA (S_1_: 212 ± 90.7 ng L^−1^) in 2007. In the case of fungicides, the highest mean concentrations were detected for penconazole (S_5_: 65.2 ± 26.9 ng L^−1^) in 2005, for cyproconazole (S_5_: 235 ± 84.6 ng L^−1^) in 2006, for penconazole (S_3_: 47.3 ± 17.6 ng L^−1^) and cyproconazole (S_4_: 44.4 ± 27.78 ng L^−1^) in 2007. Annual mean concentrations of fungicides showed an increasing trend after sampling station S_3_ which is situated after the effluent of wastewater treatment plant of Agrinio city for each separate year of sampling period. According to the results published elsewhere [25] penconazole and cyproconazole were detected in effluent samples at concentration range between <LOD and 45.08 ng L^−1^and <LOD and 349.4 ng L^−1^, respectively. The presence of azoles in water samples of Acheloos River comes from agricultural applications in the surrounding area as well as from uses for pest control in the city and also from nonagricultural sources (biocidal products used for preservation of wood and coatings). Insecticides appeared with lower concentrations in comparison with herbicides and fungicides in each sampling point, respectively. The maximum mean concentration in 2005 was detected for chlorpyrifos at S_6_ (81.79 ± 20.4 ng L^−1^), in 2006 for pirimiphos methyl at S_2_ (30.03 ± 19.7 ng L^−1^), and in 2007 for diazinon at S_5_ (45.35 ± 5.3 ng L^−1^). The different method of application among pesticide categories may have resulted in the lower levels of residual concentrations recorded in the case of insecticides.

As it can be seen in Table 2, the three-year mean concentrations along sampling stations show similar trend to annual average concentrations described above for the different pesticide groups. In the case of herbicides, the maximum three-year mean concentration was recorded for DEA (46.6–69.4 ng L^−1^) and the minimum for S-metolachlor (1.70–3.67 ng L^−1^), while there is no evidence by statistical analysis of significant differences among sampling stations for any of the target compounds. The same observation stands for fungicides with the highest three-year mean concentrations recorded for cyproconazole (14.4–92 ng/L) and the lowest for pyrazophos (1.52–3.41 ng L^−1^).

On the other hand, five of the nine detected insecticides (dichlorvos, parathion methyl, chlorpyrifos, diazinon, and fenthion) and the metabolite malaoxon had three-year mean concentrations with significant differences (*P* < 0.05) among upper (S_1_–S_3_) and lower (S_4_–S_6_) sampling stations. Diazinon had the highest mean concentration (S_5_: 26.3 ± 3.48 ng L^−1^) and dimethoate the lowest (S_1_: 2.29 ± 0.55 ng L^−1^).

#### 3.1.2. Seasonal Variation

Tables 3(a) and 3(b) present the seasonal mean concentrations of the detected compounds in river water, for each year of the sampling period. In addition Table 4 displays the three-year seasonal mean concentrations of all compounds. An overview of the results shows a seasonal variation and higher mean values for spring and summer compared to fall and winter, although the differences are not significant for all compounds either when seasonal average for each year or three-year seasonal mean concentrations are considered. It should be noticed that the application period for the detected compounds in the area generally begins in mid-March and continues until mid-July. Therefore, it was expected to record the highest concentration levels in river water during the spring-summer period.

Maximum mean values were observed in spring for the majority of the detected pesticides (nine, twelve, and ten compounds for the years 2005, 2006, and 2007, respectively). In summer, the highest mean concentration was recorded for eight compounds (malaoxon, diazinon, fenthion, pirimiphos methyl, methidathion, DEA, alachlor, and trifluralin) in 2005, five compounds (alachlor, parathion methyl, dimethoate, fenthion, and methidathion) in 2006, and four compounds (atrazine, S-metolachlor, triadimefon, and pyrazophos) in 2007. The maximum mean concentrations in autumn were recorded for pyrazophos and chloropyrifos methyl in 2005, penconazole and dichlorvos in 2006 and trifluralin, alachlor, methidathion, and S-metolachlor in 2007. Dimethoate was the only pesticide that showed a highest mean concentration in winter of 2007.

Atrazine's mean concentration, in 2005, was statistically different in spring (77.1 ± 18.7 ng L^−1^) in comparison with the other seasons. The same results were observed in the case of S-metolachlor (10.2 ± 1.26 ng L^−1^ in spring). In 2005, alachlor's mean concentration presented statistically significant difference in summer (56.5 ± 12.1 ng L^−1^) in comparison with autumn and winter. DEA presented significant differences in concentration during the spring of the year 2007 with a maximum mean concentration of 129 ± 21.9 ng L^−1^. Finally, trifluralin showed the highest mean concentration, in spring of 2006 (75.0 ± 19.6 ng L^−1^).

In the case of fungicides, mean concentrations of triadimefon (35.6 ± 3.66 ng L^−1^) and penconazole (93.0 ± 17.9 ng L^−1^) for the year 2005 were higher in spring and were significantly different in comparison with those recorded for the other seasons. In 2006, mean concentration of triadimefon (11.1 ± 2.40 ng L^−1^) and cyproconazole (282 ± 60.7 ng L^−1^) was also significantly different in spring. No significant differences for fungicides concentrations were recorded for the year 2007.

Finally in the case of insecticides, in 2005, mean concentrations of dichlorvos and parathion methyl showed higher and significantly different concentrations in spring while malaoxon and fenthion presented higher and significantly different concentrations in summer. In the spring of 2006, mean concentrations of diazinon, pirimiphos methyl, and chlorpyrifos methyl were higher and significantly different in comparison with the other seasons while dimethoate showed significantly higher concentrations in summer. For the year 2007, dichlorvos, malaoxon, and pirimiphos methyl presented higher and significantly different mean concentrations in spring.

Three of the total four fungicides (triadimefon, penconazole, and cyproconazole) showed in spring significant difference for the whole three years of sampling (2005–2007) in relation to autumn and winter (Table 4). Nonsignificant differences among seasons were observed for pyrazophos which is probably linked to its withdrawal from the market.

Most of herbicides (atrazine, trifluralin, S-metolachlor, and DEA) showed significantly higher three-year mean concentrations in spring than for the other seasons (Table 4). Methidathion and chlorpyrifos methyl are the only insecticides that did not show statistically significant differences for any of the seasons during the total sampling period. The majority of the insecticides (dichlorvos, parathion methyl, chlorpyrifos, malaoxon, and pirimiphos methyl) showed higher mean concentrations in the spring compared with the other seasons. Dimethoate and fenthion showed statistically significant difference and the highest three-year mean concentrations in summer.

In conclusion, fourteen of the nineteen compounds studied showed significant differences of three-year mean concentration values in spring and summer compared with the other two seasons (Table 4). Statistical analysis of data for the whole period of three-year sampling displays the same image in spring and summer, which were the seasons that pesticides presented the highest concentrations, since this is the main period of their application (in March until mid-July).

Table 5 presents a statistical analysis of annual mean concentrations of the analytes for the three-year monitoring. The fungicides, triadimefon, penconazole, and pyrazophos, showed the highest mean concentrations in 2005 with significant difference (*P* < 0.05) compared to 2006 and 2007. On the contrary, cyproconazole annual mean maximum value was observed in 2006. It should be noted that the compounds triadimefon and pyrazophos were exclusively applied in tobacco cultivation, which was eliminated in 2006 in the area close to the river course. As a result significant reduction in their annual mean concentrations was noticed ever since and consequently those two pesticides can be used as representative markers of changing land use [34, 35].

All the studied herbicides showed the highest mean concentration levels in 2005 with significant differences compared with 2006 and 2007, except for the metabolite desethyl atrazine (DEA) that showed the highest mean concentrations in 2007. Eight (dichlorvos, parathion methyl, chlorpyrifos, malaoxon, dimethoate, fenthion, pirimiphos methyl, and chlorpyrifos methyl) of the total ten insecticides had higher mean concentrations in 2005 with significant difference compared to 2006 or 2007. Only diazinon and methidathion showed the highest mean concentration in 2007 with significant difference in comparison with the other two sampling years and 2006, respectively.

Peak mean concentrations for twelve pesticides were observed in 2005 with a significant difference from the concentrations recorded for the other two years. Tobacco was the most important crop in Aitoloakarnania prefecture for decades with an area accounting 25% of total cultivated land. The abolition of the tobacco crop in 2006 resulted in a lower mean concentration recording of the majority of studied pesticides in river water, as 2006 was a year of fallow.

Many herbicides found in the present study in Acheloos River, such as alachlor, atrazine, S-metolachlor, trifluralin, and the metabolite deethylatrazine, as well as insecticides such as chlorpyrifos methyl, diazinon, and dimethoate, have been frequently detected in the past studies in the rivers of Greece [9, 29, 36]. Based on many previous studies [36–39], the pesticide levels in surface waters are related to the time, the way of their application, and their use in agricultural activities as well as to the physical-chemical properties of the organic compounds and of the soil and the frequency of rainfall. Important quantities of pesticides were guided from the field to the aquatic resources when the first runoff-producing rain occurred soon after application.

The highest concentrations of pesticides in surface waters surrounded by agricultural areas are dependant on meteorological and hydrological conditions. Water systems with relatively small drainage basins, such as in the case of Acheloos River, show increased pesticide concentrations with short periods of elevated concentrations, of about one month. On the other hand, lower pesticide concentration peaks were observed in autumn months after the early rainfalls following the dry summer period [9]. In winter and autumn, pesticides' mean concentration decreases considerably due to dilution effects caused by high rainfall during these seasons and the degradation that occurs after their application.

Atrazine was the most popular herbicide in Greece—especially in the cultivation of tobacco until 2006—and it was withdrawn in September 11, 2004 with the last official use in September 10, 2005. Atrazine and its metabolites can persist in water and soil for decades. Jablonowski et al. have demonstrated the high persistence of atrazine and its metabolites in soil [40, 41]. The accumulation of the parent compound in the soil may result in a long-term source of atrazine and its metabolites to ground water or surface waters [42].

To investigate the relative age of atrazine, the desethyl atrazine-to-atrazine ratio (DAR) was calculated. At first, soil microorganisms metabolize atrazine to DEA; thus, as long as atrazine remains in soil, metabolic activity continues and increasing DEA amounts were transported to surface and ground waters. DAR values greater than 1.0 are calculated for atrazine transport through the unsaturated zone, while DAR values much less than 1.0 are found when atrazine was transported off the field by surface runoff [43]. DAR values less than 0.05 were reported in runoff from agricultural areas soon after atrazine application [44] and DAR values between 0.5 and 0.7 after the atrazine application. Finally, high DAR values correspond to long periods after atrazine application in soils, while values <0.5 may suggest preferential flow in soils [45].

In our study, DAR values were quite high while most of the observed values were >1 due to the past uses of atrazine and the prolonged degradation of atrazine in soil and surface water. Garmouma et al. [46] have also reported DAR values higher than 1 for longer periods after atrazine application. During the sampling period of three years, DAR presented higher values in spring. This is in accordance with seasonal variation of triazines concentrations, which have a major input in spring after their application in crops, as reported in previous studies [47–49]. Additionally, water quantities discharging to Acheloos River due to run-off, increased also in spring. On the other hand, DAR values were lower in 2005 and increased in 2006 and the higher values were observed in 2007. Elimination of tobacco cultivation in 2006 minimized atrazine applications in the area leading thus to lower concentrations of atrazine and higher concentrations of DEA.

### 3.2. Risk Assessment

Environmental risk assessment was performed to evaluate negative impact of pesticides on the aquatic system of Acheloos River for the years 2005 and 2007. The results of calculated risk quotients for all the detected pesticides are presented in Table 6. In 2005, six pesticides (dichlorvos, chlorpyrifos, malaoxon, fenthion, pirimiphos methyl, and chlorpyrifos methyl) presented RQs higher than unit when using median MECs, while in 2007, only chlorpyrifos methyl showed RQs higher than unit. Pesticides mentioned above had the highest RQ values mainly due to their relatively high toxicity to aquatic organisms, hence producing quite low PNEC values. In addition, fungicides presented the lowest RQ values using both median and extreme MEC values for both sampling years (2005 and 2007). In general, highest RQs were calculated for insecticides, lower for herbicides, and the lowest for fungicides. This trend has been confirmed by recent studies of pesticide monitoring in drainage canals in Northeastern Greece [36]. Assessing the synergistic toxicity of pesticides in mixtures has been an enduring challenge for environmental health research and could be only determined via toxicity experiments. Based on the existing data on the combination effects from pesticide mixtures, the observed effects of pesticides from the same class are often additive [50, 51]. Even mixtures of herbicides with different modes of action generally show concentration additivity in their toxicity effects [50, 52]. For this purpose, the detected pesticides are grouped in three subcategories based on their mode of action, that is, organophosphorus insecticides, herbicides, and azole fungicides and the cumulative risk assessment is determined. Cumulative risk quotients for fungicides were always lower than unit presenting acceptable risk, while maximum cumulative RQs for herbicides were always lower than 9.3 with a decreasing trend from 2005 to 2007. The same trend was observed also for insecticides which presented the highest cumulative risks (RQ values up to 115.5 in 2005). Taking into consideration that substantial synergistic effects have been observed for insecticide mixtures [53], unacceptable risks may be suggested for organophosphorus pesticides, especially for the year 2005.

The European Union Water Framework Directive (WFD) 2000/60/EC established a framework for community action in the field of water policy that aims at reducing progressively the emission of hazardous substances and achieving a good ecological status in European river basins by 2015. A number of pesticide compounds such as alachlor, atrazine, chlorpyrifos, diuron, isoproturon, simazine, and trifluralin are included in the list of 33 priority substances defined in Annex I of the Directive 2008/105/EC. The WFD has defined the concentrations of the priority substances in water to be below the Environmental Quality Standards (EQSs), the annual average (AA), and the maximum allowed concentration (MAC). The annual average end point has been established at a level providing protection against long-term effects while the maximum allowable concentration has been established taking into consideration the protection of the aquatic ecosystem against short-term exposure [32]. It should be noted that up to six pesticides (atrazine, simazine, alachlor, trifluralin, diazinon, and chlorpyrifos) included in the list of the 33 priority substances (Annex I of the 2008/105/EC Directive) have been detected in the water samples of Acheloos River. Only chlorpyrifos and diazinon annual average concentration (in 2005 and in 2007, respectively) was slightly higher than the annual average concentration proposed by EQS (Table 7). On the other hand, the recorded pesticides' maximum concentrations were lower than the maximum allowed concentrations posed by WFD (Table 7). Thus, pesticides' monitoring results showed a good compliance with WFD for the majority of them.

## 4. Conclusions

Seventeen pesticides belonging to various categories (insecticides, herbicides, and fungicides) and two metabolites, out of thirty target compounds monitored during a three-year period (March 2005–February 2008), had detection frequencies greater than 10% in Acheloos River, one of the most important aquatic systems in Western Greece. The most frequently detected compounds were diazinon (78.6%), alachlor (50%), penconazole (43.2%), and DEA (69.3%) for insecticides, herbicides, fungicides, and metabolites categories, respectively.

The statistical analysis of the results, revealed that the spatial distribution of mean concentrations of the target compounds showed a general trend with greater values for the last three stations (S_4_, S_5_, and S_6_) than those located upstream (S_1_, S_2_, and S_3_). Seasonal variation showed in general higher mean concentrations for spring and summer compared to autumn and winter. The annual distribution of mean concentrations of pesticides was strongly affected by the elimination of tobacco cultivation in 2006. The desethyl atrazine-to-atrazine ratio (DAR) was found quite high due to the past uses of atrazine and the prolonged degradation of atrazine in soil and surface water. DAR values were lower in 2005, increased in 2006, and reached the highest values in 2007.

The environmental risk assessment of pesticide residues showed that six of the total nineteen compounds (dichlorvos, chlorpyrifos, malaoxon, fenthion, pirimiphos methyl, and chlorpyrifos methyl) presented RQs higher than unit in 2005 when using median MECs, while in 2007, only chlorpyrifos methyl showed RQs higher than unit. Fungicides presented the lowest RQ values using both median and extreme MEC values for both sampling years (2005 and 2007). Finally, the annual average (AA) and the maximum allowed concentration (MAC) of six pesticides (atrazine, simazine, alachlor, trifluralin, diazinon, and chlorpyrifos) included in the list of the 33 priority substances were in general (except chlorpyrifos for year 2005 and diazinon for year 2007) lower than the concentration levels of the Environmental Quality Standards (EQS) defined by Water Framework Directive 2000/60/EC.

## Figures and Tables

**Table 1:** Mean, minimum, and maximum concentrations (ng L^−1^) and frequency of detection (%) for the nineteen most frequently detected pesticides and metabolites.

Pesticides	Concentration (ng L^−1^)			% detection
	Mean	Min	Max	
Insecticides				
Diazinon	19.0	<LOD	70.3	78.6
Fenthion	11.1	<LOD	84.1	52.6
Methidathion	4.5	<LOD	26.7	42.2
Pirimiphos methyl	12.4	<LOD	83.5	40.1
Dichlorvos	8.0	<LOD	66.4	39.1
Malaoxon	5.5	<LOD	80.6	35.4
Chlorpyrifos	12.2	<LOD	101.2	31.8
Chlorpyrifos methyl	7.1	<LOD	77.1	31.3
Parathion methyl	6.5	<LOD	66.2	29.7
Dimethoate	3.0	<LOD	45.1	21.4

Herbicides				
Atrazine desethyl	56.1	<LOD	316.9	69.3
Atrazine	18.4	<LOD	288.3	42.7
Alachlor	18.7	<LOD	213.5	50.0
Trifluralin	14.3	<LOD	237.0	28.1
S-Metolachlor	2.0	<LOD	20.5	24.5

Fungicides				
Penconazole	21.2	<LOD	227.1	43.2
Cyproconazole	51.1	<LOD	724.1	31.8
Triadimefon	10.8	<LOD	192.3	40.1
Pyrazophos	1.4	<LOD	32.7	17.2

**Table:** (a)

		Insecticides (ng L^−1^)									
		Dichlorvos	Parathion methyl	Chlorpyrifos	Malaoxon	Dimethoate	Diazinon	Fenthion	Pirimiphos methyl	Chlorpyrifos methyl	Methidathion
2005–2007	S_1_	5.33 ± 1.77^a^	3.10 ± 1.36^a^	5.67 ± 2.4^a^	2.43 ± 0.6^a^	2.29 ± 0.55	12.8 ± 1.52^a^	4.08 ± 1.46^a^	17.5 ± 4.43	4.90 ± 1.77	2.64 ± 0.74
	S_2_	7.11 ± 2.26^a^	2.87 ± 0.94^a^	5.67 ± 2.04^a^	3.32 ± 0.8^a^	3.92 ± 1.17	17.8 ± 2.06^a,b^	7.11 ± 2.46^a,b^	12.6 ± 3.26	8.11 ± 3.31	3.41 ± 0.91
	S_3_	9.87 ± 2.66^a,b^	8.61 ± 2.84^a,b^	9.92 ± 2.86^a,b^	4.12 ± 0.9^a^	4.77 ± 1.28	18.8 ± 2.35^a,b^	11.6 ± 3.14^a,b,c^	11.2 ± 3.19	9.47 ± 3.63	4.44 ± 0.94
	S_4_	16.4 ± 3.65^b^	9.35 ± 2.91^a,b^	20.8 ± 5.70^b^	7.5 ± 2.43^a,b^	6.11 ± 1.50	24.5 ± 3.25^b^	17.6 ± 4.61^c^	14.0 ± 3.62	10.8 ± 3.31	4.24 ± 1.03
	S_5_	6.84 ± 1.90^a^	12.4 ± 2.89^b^	18.1 ± 5.57^a,b^	12.6 ± 3.69^b^	3.57 ± 0.67	26.3 ± 3.48^b^	13.6 ± 3.89^a,b,c^	11.7 ± 3.87	7.48 ± 2.51	5.83 ± 1.25
	S_6_	8.66 ± 2.88^a^	9.67 ± 2.80^a,b^	22.2 ± 6.56^b^	6.28 ± 2.15^a^	5.90 ± 1.95	23.1 ± 3.19^b^	16.0 ± 3.97^b,c^	16.9 ± 6.88	4.33 ± 1.03	4.86 ± 1.03

**Table:** (b)

		Fungicides (ng L^−1^)				Herbicides (ng L^−1^)				
		Triadimefon	Pyrazophos	Penconazole	Cyproconazole	Atrazine	Trifluralin	S-Metolachlor	DEA	Alachlor
2005–2007	S_1_	5.86 ± 1.33	1.52 ± 0.25	11.2 ± 5.11	14.4 ± 6.24	8.31 ± 1.90	5.21 ± 2.91	2.43 ± 0.69	53.3 ± 15.4	10.9 ± 2.46
	S_2_	6.81 ± 1.96	1.35 ± 0.19	14.7 ± 5.54	36.3 ± 14.04	13.9 ± 3.21	14.3 ± 7.59	3.33 ± 0.9	69.4 ± 18.1	12.2 ± 2.56
	S_3_	12.1 ± 3.42	2.36 ± 0.64	30.5 ± 10.0	65.4 ± 27.1	15.9 ± 5.14	14.7 ± 6.20	3.67 ± 0.99	46.6 ± 10.1	16.1 ± 3.25
	S_4_	18.6 ± 6.60	3.41 ± 1.28	26.4 ± 9.05	67.6 ± 22.1	30.7 ± 11.6	19.2 ± 7.02	1.7 ± 0.37	59.2 ± 13.5	20.9 ± 4.51
	S_5_	15.7 ± 3.03	3.38 ± 1.04	27.7 ± 10.1	92.0 ± 33.5	27.0 ± 10.7	15.6 ± 5.90	2.09 ± 0.67	47.9 ± 9.99	27.7 ± 8.07
	S_6_	13.7 ± 3.40	2.24 ± 0.54	24.8 ± 9.65	58.7 ± 28.6	24.3 ± 7.03	21.8 ± 8.99	2.36 ± 0.78	50.3 ± 10.3	21.3 ± 6.35

Different letters indicate significant differences at significance level *P* < 0.05 for the total sampling period of three years (2005–2007).

**Table:** (a)

		Fungicides (ng L^−1^)				Herbicides (ng L^−1^)				
		Triadimefon	Pyrazophos	Penconazole	Cyproconazole	Atrazine	Trifluralin	S-Metolachlor	DEA	Alachlor
2005	Spring	35.6 ± 3.66^c^	5.30 ± 0.57	93.0 ± 17.9^b^	16.5 ± 5.99	77.1 ± 18.7^b^	24.4 ± 3.02	10.2 ± 1.26^c^	62.2 ± 5.57^b,c^	42.8 ± 3.63^b,c^
	Summer	19.7 ± 2.00^b^	2.06 ± 0.49	39.8 ± 16.9^a^	11.7 ± 4.17	25.6 ± 5.04^a^	26.3 ± 6.44	5.46 ± 0.76^b^	90.8 ± 22.1^c^	56.5 ± 12.1^c^
	Autumn	14.1 ± 4.61^b^	6.22 ± 2.62	7.73 ± 3.1^a^	9.31 ± 4.99	29.1 ± 13.8^a^	12.2 ± 4.47	1.85 ± 0.43^a^	36.5 ± 10.9^a,b^	22.6 ± 6.82^a,b^
	Winter	1.00 ± 0.00^a^	1.00 ± 0.00	1.00 ± 0.00^a^	2.77 ± 0.77	13.9 ± 4.60^a^	19.4 ± 18.9	0.50 ± 0.00^a^	2.00 ± 0.00^a^	1.00 ± 0.00^a^

2006	Spring	11.1 ± 2.40^b^	2.70 ± 0.80	2.72 ± 1.40	282 ± 60.7^b^	24.3 ± 9.27	75.0 ± 19.6^b^	3.28 ± 1.53	39.6 ± 13.9	8.22 ± 2.13
	Summer	3.49 ± 1.20^a^	1.00 ± 0.00	2.35 ± 1.35	145 ± 38.1^a,b^	18.4 ± 7.47	0.50 ± 0.00^a^	1.88 ± 0.51	14.4 ± 3.48	8.43 ± 3.03
	Autumn	3.37 ± 0.83^a^	1.95 ± 0.44	5.47 ± 1.86	5.21 ± 0.95^a^	3.02 ± 0.94	0.50 ± 0.00^a^	0.91 ± 0.18	34.4 ± 5.46	2.13 ± 0.63
	Winter	1.38 ± 0.38^a^	1.00 ± 0.00	1.00 ± 0.00	3.53 ± 0.97^a^	1.00 ± 0.00	0.50 ± 0.00^a^	0.50 ± 0.00	8.87 ± 4.48	1.38 ± 0.38

2007	Spring	8.27 ± 3.99	1.00 ± 0.00	34.3 ± 12.83	44.9 ± 18.2	3.26 ± 1.37	1.52 ± 1.02	0.56 ± 0.06	129 ± 21.9^c^	8.56 ± 5.70
	Summer	21.6 ± 10.9	1.47 ± 0.47	26.5 ± 6.98	31.1 ± 12.5	9.83 ± 3.99	0.63 ± 0.09	0.63 ± 0.09	43.9 ± 15.2^a,b^	7.97 ± 2.07
	Autumn	2.26 ± 0.41	1.04 ± 0.04	10.8 ± 0.88	6.97 ± 2.00	4.39 ± 1.55	3.46 ± 1.54	0.63 ± 0.09	87.1 ± 26.1^b,c^	20.5 ± 3.48
	Winter	2.85 ± 1.44	1.11 ± 0.11	6.90 ± 2.01	4.30 ± 1.03	1.38 ± 0.38	1.08 ± 0.26	0.50 ± 0.00	6.17 ± 4.17^a^	10.6 ± 3.50

**Table:** (b)

		Insecticides (ng L^−1^)									
		Dichlorvos	Parathion methyl	Chlorpyrifos	Malaoxon	Dimethoate	Diazinon	Fenthion	Pirimiphos methyl	Chlorpyrifos methyl	Methidathion
2005	Spring	39.5 ± 3.43^c^	27.5 ± 3.46^b^	53.6 ± 6.37^b^	7.61 ± 0.91^a^	8.62 ± 2.42^b^	20.2 ± 1.88^b,c^	28.7 ± 5.81^b^	33.4 ± 4.44^b,c^	14.3 ± 1.4	5.77 ± 1.10^b,c^
	Summer	9.52 ± 2.01^b^	10.9 ± 3.46^a^	45.4 ± 9.06^b^	16.8 ± 3.17^b^	7.99 ± 1.26^b^	26.7 ± 2.13^c^	46.7 ± 4.98^c^	37.7 ± 9.34^c^	23.1 ± 6.64	8.63 ± 1.79^c^
	Autumn	4.18 ± 1.14^a,b^	7.08 ± 2.35^a^	18.9 ± 6.83^a^	3.57 ± 1.10^a^	1.89 ± 0.27^a^	16.9 ± 2.25^b^	10.6 ± 4.58^a^	14.5 ± 3.54^a,b^	23.6 ± 6.06	2.04 ± 0.86^a,b^
	Winter	0.50 ± 0.00^a^	1.00 ± 0.00^a^	1.00 ± 0.00^a^	1.00 ± 0.00^a^	5.90 ± 2.10^a.b^	2.00 ± 0.00^a^	0.50 ± 0.00^a^	0.50 ± 0.00^a^	0.69 ± 0.19	0.50 ± 0.00^a^

2006	Spring	1.70 ± 0.48	6.87 ± 2.37^a,b^	5.45 ± 1.54	4.70 ± 2.04	2.59 ± 0.84^a^	18.4 ± 2.58^c^	6.88 ± 2.19	31.6 ± 8.26^b^	5.70 ± 1.36^b^	1.67 ± 0.51
	Summer	1.08 ± 0.31	15.3 ± 4.33^b^	2.81 ± 1.31	1.57 ± 0.57	12.6 ± 3.20^b^	7.81 ± 1.32^a,b^	8.87 ± 2.02	1.22 ± 0.45^a^	2.26 ± 0.81^a^	3.99 ± 1.38
	Autumn	1.75 ± 0.36	4.37 ± 1.04^a^	3.50 ± 1.07	2.41 ± 0.59	1.89 ± 0.27^a^	10.3 ± 1.10^b^	5.34 ± 1.44	0.69 ± 0.10^a^	0.69 ± 0.10^a^	2.45 ± 0.68
	Winter	1.21 ± 0.51	1.00 ± 0.00^a^	1.95 ± 0.95	1.22 ± 0.14	3.07 ± 1.32^a^	3.70 ± 1.15^a^	1.12 ± 0.62	0.88 ± 0.24^a^	0.50 ± 0.00^a^	0.50 ± 0.00

2007	Spring	21.6 ± 3.73^b^	1.33 ± 0.33	3.76 ± 1.86	19.2 ± 6.36^b^	1.51 ± 0.40^a^	36.2 ± 3.52^b^	1.84 ± 1.34^a,b^	16.4 ± 4.81^b^	2.58 ± 1.09	1.30 ± 0.80
	Summer	5.80 ± 2.73^a^	1.13 ± 0.13	1.11 ± 0.06	1.71 ± 0.52^a^	1.34 ± 0.34^a^	33.4 ± 4.61^b^	0.66 ± 0.16^a^	3.11 ± 0.62^a^	0.76 ± 0.12	3.30 ± 1.45
	Autumn	4.33 ± 1.64^a^	1.26 ± 0.18	1.29 ± 0.08	1.43 ± 0.07^a^	1.00 ± 0.00^a^	32.5 ± 3.71^b^	4.48 ± 0.72^b^	0.56 ± 0.06^a^	1.27 ± 0.13	10.2 ± 1.15
	Winter	0.99 ± 0.30^a^	1.00 ± 0.00	1.11 ± 0.11	2.17 ± 0.92^a^	5.48 ± 3.27^b^	3.60 ± 1.60^a^	4.67 ± 1.35^b^	0.50 ± 0.00^a^	1.07 ± 0.26	8.05 ± 0.90

Different letters indicate significant differences at significance level *P* < 0.05 for each year separately of the total sampling period of three years (2005–2007).

**Table:** (a)

		Fungicides (ng L^−1^)				Herbicides (ng L^−1^)				
		Triadimefon	Pyrazophos	Penconazole	Cyproconazole	Atrazine	Trifluralin	S-Metolachlor	DEA	Alachlor
2005–2007	Spring	18.3 ± 2.57^c^	3.00 ± 0.40	43.3 ± 8.86^b^	114 ± 26.5^b^	34.9 ± 8.07^b^	33.6 ± 7.74^b^	4.70 ± 0.86^c^	77.2 ± 10.1^b^	19.8 ± 3.21^b^
	Summer	14.9 ± 3.81^b,c^	1.51 ± 0.23	22.8 ± 6.38^a,b^	62.9 ± 15.5^a,b^	17.9 ± 3.34^a,b^	9.14 ± 2.69^a^	2.65 ± 0.41^b^	49.7 ± 9.84^b^	24.3 ± 5.18^b^
	Autumn	6.58 ± 1.70^a,b^	2.96 ± 0.84	7.99 ± 1.26^a^	7.16 ± 1.80^a^	12.2 ± 4.84^a^	5.37 ± 1.69^a^	1.13 ± 0.17^a,b^	52.6 ± 9.98^b^	15.1 ± 2.81^b^
	Winter	1.74 ± 0.51^a^	1.04 ± 0.04	2.97 ± 0.92^a^	3.53 ± 0.53^a^	5.43 ± 2.05^a^	7.00 ± 6.30^a^	0.50 ± 0.00^a^	5.68 ± 2.03^a^	4.32 ± 1.54^a^

**Table:** (b)

		Insecticides (ng L^−1^)									
		Dichlorvos	Parathion methyl	Chlorpyrifos	Malaoxon	Dimethoate	Diazinon	Fenthion	Pirimiphos methyl	Chlorpyrifos methyl	Methidathion
2005–2007	Spring	20.9 ± 2.70^b^	11.9 ± 2.43^c^	20.9 ± 3.88^c^	10.5 ± 2.37^b^	4.24 ± 0.95^a,b^	24.9 ± 1.9^b^	12.5 ± 2.62^b,c^	27.2 ± 3.60^c^	7.53 ± 1.00	2.91 ± 0.55
	Summer	5.47 ± 1.21^a^	9.11 ± 1.99^b,c^	16.4 ± 4.11^b,c^	6.68 ± 1.45^a,b^	7.31 ± 2.13^b^	22.6 ± 2.27^b^	18.7 ± 3.27^c^	14.0 ± 3.83^b^	8.71 ± 2.60	5.31 ± 0.94
	Autumn	3.42 ± 0.68^a^	4.24 ± 0.9^a,b^	7.91 ± 2.50^a,b^	2.47 ± 0.43^a^	1.60 ± 0.14^a^	19.9 ± 1.94^b^	6.81 ± 1.63^a,b^	5.26 ± 1.47^a,b^	8.53 ± 2.47	4.90 ± 0.73
	Winter	0.90 ± 0.20^a^	1.00 ± 0.00^a^	1.35 ± 0.32^a^	1.46 ± 0.31^a^	4.82 ± 1.32^a,b^	3.10 ± 0.65^a^	2.09 ± 0.64^a^	0.63 ± 0.09^a^	0.76 ± 0.12	3.02 ± 0.91

Different letters indicate significant differences at significance level *P* < 0.05 for the total sampling period of three years (2005–2007).

**Table:** (a)

	Fungicides (ng L^−1^)				Herbicides (ng L^−1^)				
	Triadimefon	Pyrazophos	Penconazole	Cyproconazole	Atrazine	Trifluralin	S-Metolachlor	DEA	Alachlor
2005	20.9 ± 2.31^b^	4.07 ± 0.78^b^	42.3 ± 8.69^c^	11.5 ± 2.65^a^	40.9 ± 7.68^b^	20.8 ± 3.12^b^	5.32 ± 0.65^b^	57.1 ± 8.26^b^	36.7 ± 4.81^b^
2006	5.54 ± 0.96^a^	1.79 ± 0.29^a^	3.26 ± 0.81^a^	130 ± 25.9^b^	13.8 ± 3.72^a^	22.8 ± 7.28^b^	1.87 ± 0.50^a^	27.4 ± 4.79^a^	5.77 ± 1.18^a^
2007	9.91 ± 3.57^a^	1.16 ± 0.14^a^	22.1 ± 4.52^b^	25.3 ± 6.87^a^	5.38 ± 1.38^a^	1.79 ± 0.56^a^	0.60 ± 0.04^a^	78.8 ± 12.2^b^	12.2 ± 2.20^a^

**Table:** (b)

	Insecticides (ng L^−1^)									
	Dichlorvos	Parathion methyl	Chlorpyrifos	Malaoxon	Dimethoate	Diazinon	Fenthion	Pirimiphos methyl	Chlorpyrifos methyl	Methidathion
2005	16.0 ± 2.37^c^	13.7 ± 2.31^c^	35.5 ± 4.49^b^	8.48 ± 1.27^b^	6.14 ± 0.91^b^	19.4 ± 1.40^b^	28.9 ± 3.37^b^	25.8 ± 3.62^b^	18.4 ± 2.83^b^	4.98 ± 0.77^b^
2006	1.48 ± 0.21^a^	8.05 ± 1.62^b^	3.73 ± 0.70^a^	2.73 ± 0.67^a^	5.44 ± 1.16^b^	11.3 ± 1.12^a^	6.44 ± 1.02^a^	10.1 ± 3.04^a^	2.65 ± 0.54^a^	2.48 ± 0.5^a^
2007	9.61 ± 1.78^b^	1.21 ± 0.12^a^	1.96 ± 0.57^a^	6.93 ± 2.15^b^	1.70 ± 0.38^a^	31.0 ± 2.36^c^	2.56 ± 0.52^a^	6.08 ± 1.68^a^	1.48 ± 0.34^a^	5.24 ± 0.77^b^

Different letters indicate significant differences at significance level *P* < 0.05 for each year separately of the total sampling period of three years (2005–2007).

**Table 6:** Environmental risk of pesticides detected in Acheloos River as risk quotient (MEC/PNEC) based on median and maximum residue values for 2005 and 2007.

Pesticide	PNEC (*μ*g L^−1^)	Assessment factor	RQ_median_	RQ_max_	RQ_median_	RQ_max_
			2005		2007	
Dichlorvos	0.0038	50	2.21	17.47	0.53	12.63
Parathion methyl	0.0073	1000	0.25	9.07	0.27	0.96
Triadimefon	0.34	50	0.05	0.22	0.01	0.57
Chlorpyrifos	0.014	10	1.71	7.23	0.14	2.41
S-metolachlor	15.6	50	0.0003	0.001	0.0001	0.0001
Malaoxon	0.003	50	1.90	17.83	0.67	26.87
Pyrazophos	0.0036	50	0.28	9.08	0.28	2.61
Dimethoate	4	10	0.001	0.01	0.001	0.01
Diazinon	0.056	10	0.36	0.73	0.53	1.26
Penconazole	1.2	50	0.002	0.19	0.01	0.15
Fenthion	0.0057	1000	3.33	14.75	0.18	4.33
Atrazine desethyl	0.72	1000	0.06	0.44	0.05	0.44
Alachlor	2	10	0.02	0.11	0.002	0.05
Cyproconazole	2	10	0.002	0.04	0.002	0.12
Atrazine	10	10	0.003	0.03	0.0002	0.01
Trifluralin	0.5	10	0.04	0.23	0.01	0.04
Pirimiphos methyl	0.0016	50	16.28	50.67	0.63	41.15
Chlorpyrifos methyl	0.001	10	10.00	77.11	1.08	19.61
Methidathion	0.0132	50	0.15	2.02	0.15	1.90

**Table 7:** Compliance of detected concentrations with environmental quality standards (EQS) (*μ* L^−1^) established for the priority pesticides monitored in the present study.

Pesticide	AA-EQS	MAC-EQS	AA-River			MAC-River		
	Inland waters	Inland waters	Acheloos			Acheloos		
			2005	2006	2007	2005	2006	2007
Alachlor	0.3	0.7	0.037	0.006	0.012	0.213	0.039	0.098
Atrazine	0.6	2	0.041	0.014	0.005	0.288	0.138	0.068
Chlorpyrifos	0.03	0.1	0.035	0.004	0.002	0.101	0.024	0.034
Diazinon	0.02	—	0.019	0.011	0.031	0.041	0.039	0.070
Simazine	1	4	Sporadically detections			Sporadically detections		
Trifluralin	0.03	Not applicable	0.021	0.023	0.002	0.115	0.237	0.031
